# Genomics and prevalence of bacterial and archaeal isolates from biogas-producing microbiomes

**DOI:** 10.1186/s13068-017-0947-1

**Published:** 2017-11-13

**Authors:** Irena Maus, Andreas Bremges, Yvonne Stolze, Sarah Hahnke, Katharina G. Cibis, Daniela E. Koeck, Yong S. Kim, Jana Kreubel, Julia Hassa, Daniel Wibberg, Aaron Weimann, Sandra Off, Robbin Stantscheff, Vladimir V. Zverlov, Wolfgang H. Schwarz, Helmut König, Wolfgang Liebl, Paul Scherer, Alice C. McHardy, Alexander Sczyrba, Michael Klocke, Alfred Pühler, Andreas Schlüter

**Affiliations:** 1 Center for Biotechnology (CeBiTec), Bielefeld University, Universitätsstrasse 27, 33615 Bielefeld, Germany; 2 Faculty of Technology, Bielefeld University, Universitätsstrasse 25, 33615 Bielefeld, Germany; 3 Computational Biology of Infection Research, Helmholtz Centre for Infection Research, Inhoffenstraße 7, 38124 Brunswick, Germany; 4 German Center for Infection Research (DZIF), partner site Hannover-Braunscheig, Inhoffenstraße 7, 38124 Brunswick, Germany; 5 Department Bioengineering, Leibniz Institute for Agricultural Engineering and Bioeconomy (ATB), Max-Eyth-Allee 100, 14469 Potsdam, Germany; 6 Johannes Gutenberg-University, Institute of Microbiology and Wine Research, Johann-Joachim Becherweg 15, 55128 Mainz, Germany; 7 Department of Microbiology, Technische Universität München, Emil-Ramann-Str. 4, 85354 Freising-Weihenstephan, Germany; 8 Faculty Life Sciences/Research Center ‘Biomass Utilization Hamburg’, University of Applied Sciences Hamburg (HAW), Ulmenliet 20, 21033 Hamburg-Bergedorf, Germany; 9 Institute of Molecular Genetics, Russian Academy of Science, Kurchatov Sq. 2, Moscow, 123182 Russia; 10 Institut für Forensische Genetik GmbH, Im Derdel 8, 48168 Münster, Germany

**Keywords:** Anaerobic digestion, Biomethanation, Genome sequencing, Fragment recruitment, *Defluviitoga tunisiensis*, *Methanoculleus bourgensis*

## Abstract

**Background:**

To elucidate biogas microbial communities and processes, the application of high-throughput DNA analysis approaches is becoming increasingly important. Unfortunately, generated data can only partialy be interpreted rudimentary since databases lack reference sequences.

**Results:**

Novel cellulolytic, hydrolytic, and acidogenic/acetogenic *Bacteria* as well as methanogenic *Archaea* originating from different anaerobic digestion communities were analyzed on the genomic level to assess their role in biomass decomposition and biogas production. Some of the analyzed bacterial strains were recently described as new species and even genera, namely *Herbinix hemicellulosilytica* T3/55^T^, *Herbinix luporum* SD1D^T^, *Clostridium bornimense* M2/40^T^, *Proteiniphilum saccharofermentans* M3/6^T^, *Fermentimonas caenicola* ING2-E5B^T^, and *Petrimonas mucosa* ING2-E5A^T^. High-throughput genome sequencing of 22 anaerobic digestion isolates enabled functional genome interpretation, metabolic reconstruction, and prediction of microbial traits regarding their abilities to utilize complex bio-polymers and to perform specific fermentation pathways. To determine the prevalence of the isolates included in this study in different biogas systems, corresponding metagenome fragment mappings were done. *Methanoculleus bourgensis* was found to be abundant in three mesophilic biogas plants studied and slightly less abundant in a thermophilic biogas plant, whereas *Defluviitoga tunisiensis* was only prominent in the thermophilic system. Moreover, several of the analyzed species were clearly detectable in the mesophilic biogas plants, but appeared to be only moderately abundant. Among the species for which genome sequence information was publicly available prior to this study, only the species *Amphibacillus xylanus*, *Clostridium clariflavum*, and *Lactobacillus acidophilus* are of importance for the biogas microbiomes analyzed, but did not reach the level of abundance as determined for *M. bourgensis* and *D. tunisiensis*.

**Conclusions:**

Isolation of key anaerobic digestion microorganisms and their functional interpretation was achieved by application of elaborated cultivation techniques and subsequent genome analyses. New isolates and their genome information extend the repository covering anaerobic digestion community members.

**Electronic supplementary material:**

The online version of this article (10.1186/s13068-017-0947-1) contains supplementary material, which is available to authorized users.

## Background

Anaerobic digestion (AD) and biomethanation are commonly applied for the treatment and decomposition of organic material and bio-waste, finally yielding methane (CH_4_)-rich biogas. The whole AD process can be divided into four phases: hydrolysis, acidogenesis, acetogenesis, and methanogenesis. Organic polymers are hydrolyzed into sugar molecules, fatty acids, and amino acids by hydrolytic enzymes. These metabolites are further degraded into the intermediate volatile fatty acids (VFA), acetate, alcohols, carbon dioxide (CO_2_), and hydrogen (H_2_) during acidogenesis and acetogenesis. Finally, CH_4_ is produced either from acetate or from H_2_ and CO_2_. The challenges in each of these steps are reflected within the complexity of the microbial community converting biomass to biogas. Community compositions and dynamics were frequently investigated using different molecular biological methods. Among these, quantitative ‘real-time’ polymerase chain reaction (qPCR), e.g., [1–5], terminal restriction fragment length polymorphism (TRFLP) [6–8], and the 16S rRNA gene amplicon [9, 10] as well as metagenome sequencing approaches [9, 11–14] applying high-throughput (HT) technologies are the most commonly used methods. In these studies, bacterial members belonging to the classes *Clostridia* and *Bacteroidia* were identified to dominate the biogas microbial communities, followed by *Proteobacteria*, *Bacilli*, *Flavobacteria*, *Spirochaetes*, and *Erysipelotrichi*. Within the domain *Archaea*, members from the orders *Methanomicrobiales*, *Methanosarcinales*, and *Methanobacteriales* were described to be abundant in biogas systems.

However, all recently published metagenome and metatranscriptome studies addressing elucidation of the biogas microbiology reported on a huge fraction of unassignable sequences suggesting that most of the microorganisms in biogas communities are so far unknown [15–18]. This is due to the limiting availability of reference strains and their corresponding genome sequences in public databases. Moreover, reference sequences are often derived from only distantly related strains isolated from different environments. For a better understanding of the microbial trophic networks in AD and any further biotechnological optimization of the biomethanation process, extension of public databases regarding relevant sequence information seems to be an indispensable prerequisite.

Recently, studies on the isolation, sequencing, and physiological characterization of novel microbial strains from various mesophilic and thermophilic biogas reactors were published, e.g., [18–29]. However, only few of these studies addressed the question of whether the described strain played a dominant role within the analyzed microbial community. Accordingly, the objective of this work was to sequence and analyze a collection of recently described as well as newly isolated bacterial and archaeal strains from different biogas microbial communities to provide insights into their metabolic potential and life-style, and to estimate their prevalence in selected agricultural biogas reactors. In total, 22 different strains originating from meso- and thermophilic anaerobic digesters utilizing renewable primary products and/or organic wastes were analyzed. Based on genome analyses, isolates were functionally classified and assigned to functional roles within the AD process. Moreover, refinement of the metagenome fragment recruitment approach was used for the evaluation of an isolate’s prominence in different biogas communities. Overall the aim of this study was the considerable complementation of the reference repository by new genome information regarding AD communities.

## Methods

### Microbial strains used in this study and isolation of novel strains

In this study, 22 bacterial and archaeal strains were studied from eight meso- and thermophilic, laboratory-scale and agricultural biogas plants (BGPs) utilizing renewable primary products as well as from three further AD sources (detailed information listed in Table 1). The strains *Methanoculleus chikugoensis* L21-II-0 and *Sporanaerobacter* sp. PP17-6a were isolated within this study as follows.

**Table 1:** Summary of 22 bacterial and archaeal strains used in this study

Species and strain	Family	Origin					Reference for the isolation strategy or strain origin	Closest related NCBI GenBank entry with a validly published taxonomic affiliation	Similarity of 16S rRNA gene between isolate and GenBank entry (%)	NCBI GenBank entry of closest relative
		Location of BGP		Type of reactor	Fed substrate	T (°C) of reactor				
		Latitude	Longitude							
*Bacteria*										
*Clostridium cellulosi* DG5	*Clostridiaceae*	51.255499	6.396524	Liquid pump/wet fermentation	Maize, pig manure, grass	54	[18]^b^	*Clostridium cellulosi* AS1.1777	98.8	LN881577
*Clostridium* sp. N3C		51.255499	6.396524	Liquid pump/wet fermentation	Maize, pig manure, grass	54	[18]^c^	*Clostridium putrefaciens* DSM 1291^T^	93.0	NR113324
*Clostridium bornimense* M2/40^T^		52.3871	13.0993	Lab-scale UASS/wet fermentation	Maize silage, wheat straw	37	[20]	*Clostridium bornimense* M2/40^T^	100	JQ388596
*Clostridium thermocellum* BC1		48.135125	11.581981	Bio-waste compost treatment site close to BGP		60	[18]^d^	*Clostridium thermocellum* DSM 1237^T^	99.0	NR074629
*Proteiniborus* sp. DW1	*Clostridiales incertae sedis*	49.512893	7.083068	CSTR, wet fermentation	Maize silage, grass, cattle manure	39	[21]	*Proteiniborus ethanoligenes* GW^T^	96.0	NR044093
*Sporanaerobacter* sp. PP17-6a		51.255499	6.396524	Lab-scale CSTR/wet fermentation	Maize silage, pig manure, cattle manure	37	This study	*Sporanaerobacter acetigenes* Lup33	91.0	NR025151
*Herbinix hemicellulosilytica* T3/55^T^	*Lachnospiraceae*	51.255499	6.396524	Liquid pump/wet fermentation	Maize, pig manure, grass	54	[18, 54]^b^	*Herbinix hemicellulosilytica* T3/55^T^	100	LN626355
*Herbinix luporum* SD1D^T^		51.255499	6.396524	Liquid pump/wet fermentation	Maize, pig manure, grass	54	[18, 55]^b^	*Herbinix luporum* SD1D^T^	100	LN626359
*Peptoniphilaceae* bacterium str. ING2-D1G	*Peptoniphilaceae*	51.255499	6.396524	Lab-scale CSTR/wet fermentation	Maize silage, pig manure, cattle manure	37	[22]	*Peptoniphilus indolicus* DSM 20464^T^	90.6	AY153431
*Propionispora* sp. 2/2-37	*Veillonellaceae*	48.3924	11.7569	CSTR, wet fermentation	Maize silage, grass	38	[18]^e^	*Propionispora hippei* KS^T^	95.0	NR036875
*Bacillus thermoamylovorans* 1A1	*Bacillaceae*	48.3924	11.7569	CSTR, wet fermentation	Maize silage, pig manure	52	[18]^f^	*Bacillus thermoamylovorans* DKP^T^	99.0	NR029151
*Proteiniphilum saccharofermentans* M3/6^T^	*Porphyromonadaceae*	52.3871	13.0993	Lab-scale UASS/wet fermentation	Maize silage, wheat straw	37	[26]	*Proteiniphilum saccharofermentans* M3/6^T^	100	KP233809
*Fermentimonas caenicola* ING2-E5B^T^		51.255499	6.396524	Lab-scale CSTR/wet fermentation	Maize silage, pig manure, cattle manure	37		*Fermentimonas caenicola* ING2-E5B^T^	100	KP233810
*Petrimonas mucosa* ING2-E5A^T^		51.255499	6.396524	Lab-scale CSTR/wet fermentation	Maize silage, pig manure, cattle manure	37		*Petrimonas mucosa* ING2-E5A^T^	100	KP233808
*Defluviitoga tunisiensis* L3	*Petrotogaceae*	51.255499	6.396524	Liquid pump/wet fermentation	Maize, pig manure, grass	54	[27]	*Defluviitoga tunisiensis* SulfLac1^T^	99.9	NR122085
*Archaea*										
*Methanobacterium formicicum* MF^T^	*Methanobacteriaceae*	DSMZ^a^				37	[50]	*Methanobacterium formicicum* MF^T^	100	NR115168
*Methanobacterium formicicum* Mb9		49.878359	6.481390	CSTR, wet fermentation	Maize silage, grass, cattle manure	40	[21]	*Methanobacterium formicicum* MF^T^	100	NR115168
*Methanobacterium* sp. Mb1		49.512893	7.083068	CSTR, wet fermentation	Maize silage, grass, cattle manure	39		*Methanobacterium formicicum* MF^T^	98.0	NR115168
*Methanobacterium congolense* Buetzberg		53.736687	10.083949	CSTR, dry fermentation	Household garbage	37	[18]^g^	*Methanobacterium congolense* C^T^	99.0	NR028175
*Methanothermobacter wolfeii* SIV6		51.255499	6.396524	Liquid pump/wet fermentation	Maize, pig manure, grass	54	[18]^h^	*Methanothermobacter wolfeii* VKM B-1829^T^	100	NR040964.1
*Methanoculleus bourgensis* MS2^T^	*Methanomicrobiaceae*	DSMZ				37	[49]	*Methanoculleus bourgensis* MS2^T^	100	NR042786
*Methanoculleus chikugoensis* L21-II-0		51.255499	6.396524	Lab-scale CSTR/wet fermentation	Maize silage, pig manure, cattle manure	37	This study	*Methanoculleus chikugoensis* MG62^T^	99.0	NR028152

CSTR, continuously stirred tank reactor; UASS, upflow anaerobic solid-state reactor

^a^DSMZ, Leibniz Institute DSMZ-German Collection of Microorganisms and Cell Cultures, Braunschweig, Germany

^b^Isolation strategy number four described in more detail by [18]

^c^Isolation strategy number eight (a) published in [18]

^d^Isolation strategy number five published in [18]

^e^Isolation strategy number seven published in [18]

^f^Isolation strategy number two published in [18]

^g^Isolation strategy number ten published in [18]

^h^Isolation strategy number eleven published in [18]

Methanoculleus chikugoensis L21-II-0 Reactor material was diluted fivefold in DSMZ medium 287 [30] containing 20 mM acetate and H_2_/CO_2_ as the only carbon and energy sources. Initial incubation occurred at 37 °C for 4 weeks without antibiotics. Subsequent cultivation was performed by successive transfer of culture aliquots after incubation periods of 4 weeks into the same medium supplemented with different combinations of the antibiotics tetracycline HCl (15 µg ml^−1^), vancomycin HCl (50 µg ml^−1^), ampicillin (100 µg ml^−1^), and bacitracin (15 µg ml^−1^) or with penicillin (350 µg ml^−1^). After a total of 12 cultivation cycles, purity of the culture was confirmed by microscopic inspection and by denaturing gradient gel electrophoresis (DGGE) fingerprint analysis. Strain *M. chikugoensis* L21-II-0 is available from the Leibniz Institute German Collection of Microorganisms and Cell Cultures (DSMZ, Braunschweig, Germany) under the Accession No. DSM 100195. *Sporanaerobacter* sp. PP17-6a: Reactor material was diluted 5 × 10^6^-fold in DSMZ medium 120 [31]. After 4 weeks of incubation at 37 °C, an aliquot of the culture was transferred into the same medium supplemented with penicillin (350 µg ml^−1^). Transfer and incubation in the same medium were repeated four times. Subsequently, cultivation occurred by successive transfer of culture aliquots after incubation periods of 4 weeks into fresh medium supplemented with different combinations of antibiotics as mentioned above for isolation of the strain L21-II-0. After 14 cultivation cycles, isolation of the bacterial strain was performed by plating of the culture material on BBL™ Columbia Agar Base medium (Th. Geyer, Germany) supplemented with 5% laked horse blood (Oxoid, Germany). For purification, single colonies were picked and re-streaked, and incubation occurred at 37 °C.

### Phylogenetic classification of the analyzed bacterial and archaeal strains

To determine the phylogenetic relationship between the different strains and closely related type strains, a phylogenetic tree was constructed. For this, the 16S rRNA gene sequences retrieved from the genome sequences of the analyzed strains were aligned using the SINA alignment service v.1.2.11, which is provided online [32]. Subsequently, the SINA alignment and the All-Species Living Tree LTPs123 [33] from the SILVA ribosomal RNA project [34], only consisting of the 16S rRNA gene sequences of validly described type strains, were loaded into the ARB program [35]. Finally, the SINA alignment was placed into the existing LTP tree using ARB’s parsimony method. Only type strains closely related to the corresponding isolate analyzed within this study are shown in the tree, whereas the remaining type strains were hidden manually applying “remove species from the tree” function implemented in ARB.

### Genomic DNA extraction, sequencing, and bioinformatic analyses of biogas community members

Whole genome sequences of 13 strains, which were used in this study, were published previously (references given in Table 2). Genome sequencing of the following strains was performed within this study: *Proteiniborus* sp. DW1, *Clostridium* sp. N3C (DSM 100067), *Sporanaerobacter* sp. PP17-6a, *Proteiniphilum saccharofermentans* M3/6^T^, *Petrimonas mucosa* ING2-E5A^T^, *Methanobacterium formicicum* Mb9, *Methanobacterium congolense* Buetzberg, [36] *Methanothermobacter wolfeii* SIV6, and *M. chikugoensis* L21-II-0. In the case of *Clostridium* sp. N3C, *Sporanaerobacter* sp. PP17-6a, and *P. saccharofermentans* M3/6^T^, genomic DNA was extracted applying the innuPREP Bacteria DNA Kit (Analytik Jena, Germany). Genomic DNA of *P. mucosa* ING2-E5A^T^ and *M. chikugoensis* L21-II-0 was extracted as described previously [37]. Genomic DNA of the strain *Proteiniborus* sp. DW1 was obtained applying the protocol published previously [19] and genomic DNA from *M. congolense* Buetzberg was extracted from 10 × 10 ml of a liquid culture using the Gene Matrix stool DNA purification kit (Roboklon, Germany). DNA of strain *M. wolfeii* SIV6 was obtained applying the FastDNA Spin Kit for Soil (MP Biomedicals).

**Table 2:** Genome features of 22 bacterial and archaeal strains used in this study

Species and strain	Assembly status		Genome size (bp)	GC content (%)	No. of genes	No. of *rrn* operons	No. of tRNA genes	No. of protein coding genes	EBI accession no.	References
	Genome structure	No. of contigs								
*Bacteria*										
*Clostridium cellulosi* DG5	CCC	n.a.	2,229,578	44.15	2088	6	59	2017	ERP006074	[53]
*Clostridium* sp. N3C	Draft genome	109	3,037,440	32.43	2880	3	66	2880	FMJL01000001–FMJL01000109	This study
*Clostridium bornimense* M2/40^T^	CCC	n.a.	2,917,864	29.78	2694	8	56	2613	HG917868	[37]
	Chromid		699,161	28.09	680	0	0	680	HG917869	
*Clostridium thermocellum* BC1	Draft genome	139	3,454,918	39.10	3094	4	52	3095	CBQ0010000001–CBQ0010000139	[61]
*Proteiniborus* sp. DW1^a^	Draft genome	62	3,121,392	32.44	2795	3	40	1793	FMDO01000001–FMDO01000062	This study
*Sporanaerobacter* sp. PP17-6a	Draft genome	53	3,296,672	33.45	3148	1	46	3148	FMIF01000001–FMIF01000053	This study
*Herbinix hemicellulosilytica* T3/55^T^	Draft genome	35	3,037,031	36.69	2681	4	35	1726	CVTD020000001–CVTD020000035	[24]
*Herbinix luporum* SD1D^T^	CCC	n.a.	2,609,352	35.25	2362	4	53	1517	LN879430	[78]
*Peptoniphilaceae* bacterium str. ING2-D1G	CCC	n.a.	1,601,846	34.85	1541	4	53	1476	LM997412	[22]
*Propionispora* sp. 2/2–37	Draft genome	43	4,122,013	45.58	3690	1	76	2685	CYSP01000001–CYSP01000043	[29]
*Bacillus thermoamylovorans* 1A1	Draft genome	106	3,708,331	37.28	3472	10	59	2957	CCRF01000001–CCRF01000106	[79]
*Proteiniphilum saccharofermentans* M3/6^T^	CCC	n.a.	4,414,963	43.63	3450	3	48	3447	LT605205	This study
*Fermentimonas caenicola* ING2-E5B^T^	CCC	n.a.	2,808,926	37.30	2455	2	44	2405	LN515532	[25]
*Petrimonas mucosa* ING2-E5A^T^	CCC	n.a.	3,362,317	48.24	2693	2	46	2693	ERS1319466	This study
*Defluviitoga tunisiensis* L3	CCC	n.a.	2,053,097	31.38	1881	3	47	1815	LN824141	[23]
*Archaea*										
*Methanobacterium formicicum* MF^T^	CCC	n.a.	2,478,074	41.23	2409	2	44	2100	LN515531	[80]
*Methanobacterium formicicum* Mb9	CCC	n.a.	2,494,510	41.14	2416	2	43	2126	ERS549551	This study
*Methanobacterium* sp. Mb1	CCC	n.a.	2,029,766	39.74	2021	2	41	1689	HG425166	[19]
*Methanobacterium congolense* Buetzberg	CCC	n.a.	2,459,553	38.48	2351	3	41	2351	LT607756	This study
	Plasmid		18,118	36.05	24	0	0	24	LT607757	
*Methanothermobacter wolfeii* SIV6	CCC	n.a.	1,686,891	48.89	1793	2	36	1444	ERS1319767	This study
*Methanoculleus bourgensis* MS2^T^	CCC	n.a.	2,789,773	60.64	2586	1	45	2586	HE964772	[81]
*Methanoculleus chikugoensis* L21-II-0	Draft genome	70	2,649,997	61.83	2671	1	45	2671	FMID01000001–FMID01000070	This study

CCC, circulary closed chromosome; n.a., not applicable

^a^The strain *Proteiniborus* sp. DW1 was cultivated together with *Methanobacterium* sp. Mb1; the DW1 genome sequence was recovered from sequencing of a mixed culture consisting of strains DW1 and Mb1

For bacterial strains mentioned above, 4 μg of purified chromosomal DNA was used to construct an 8-k mate-pair sequencing library (Nextera Mate Pair Sample Preparation Kit, Illumina Inc., Eindhoven, Netherlands) and sequenced applying the mate-pair protocol on an Illumina MiSeq system. Sequencing libraries of the archaeal strains *M. chikugoensis* L21-II-0 and *M. wolfeii* SIV6 were made from 2 µg of chromosomal DNA using the TruSeq DNA PCR-Free Library Preparation Kit (Illumina Inc., Eindhoven, Netherlands) and sequenced applying the paired-end protocol on an Illumina MiSeq system.

The obtained sequences were de novo assembled using the GS de novo Assembler Software (version 2.8, Roche). An in silico gap closure approach was performed [38], which resulted in a draft genome sequence or in a circular chromosome. Gene prediction and annotation of the genomes were performed within the GenDB 2.0 annotation system [39]. Manual metabolic pathway reconstruction was carried out by means of the KEGG pathway mapping implemented in GenDB that compares gene sequences with the corresponding gene product sequences of the NCBI database, with pairwise protein sequence identity being at least 30%. To predict genes encoding carbohydrate-active enzymes, the carbohydrate-active enzyme database (CAZy) annotation web-server dbCAN [40] was used.

### Prevalence of the investigated strains within microbial communities of four different agricultural biogas plants applying the metagenome fragment recruitment approach

To evaluate the prevalence of the 22 analyzed strains within the microbial communities of the four different BGPs described previously [41], the corresponding metagenome sequences available for these BGPs (metagenome Accession Nos. at the NCBI database: SRA357208-09, SRA357211, SRA357213-14, SRA357221-23) were mapped on the genome sequences of these isolates with FR-HIT (v0.7; [42]) to sensitively recruit also metagenomic reads with lower sequence identity (global alignment down to 75% nucleotide sequence identity; Additional file 1).

As a baseline to compare against, four known and abundant metagenome-assembled genomes (MAGs) published previously [41] were included (the fifth genome bin 206_*Thermotogae* matching *Defluviitoga tunisiensis* L3 was excluded, because it is contained in the isolate collection; Table 1).

Furthermore, Mash (v1.1; [43]) was used to quickly identify potentially abundant and publicly available genome sequences in RefSeq (as of June 14, 2016; [44]). The meaning of abundance in this context refers exclusively to the number of metagenome sequences mapped to the genome sequence. For a sketch size of 1,000,000 and a k-mer size of 21, pairwise distances between the metagenomic read sets and all 5061 genomes in RefSeq (plus, as a control, the 22 strains from this study) were calculated. Requiring a minimum of 20 k-mer hits not only confirmed the potential relevance of the selected 22 strains, but additionally identified 46 publicly available strains from RefSeq for further analyses.

All metagenome sequences available for the four BGPs were mapped on the genome sequences of these isolates, the four MAGs, and the 46 reference strains with Kallisto [45] (v0.43.1). For each genome, the GPM (genomes per million) values were calculated using the TPM (transcripts per million) values reported by Kallisto (see Additional file 3).

## Results and discussion

### Selection of a set of microbial isolates from different biogas-producing communities

Limited availability of genome sequence information in public databases for AD community members generally constrains the interpretation of metagenomic and metatranscriptomic data of such communities leading to large amounts of non-classifiable metagenome sequences from AD habitats [15–18, 46, 47]. Accordingly, parallel application of both traditional culturomics [48] as well as molecular analysis combined with HT sequencing techniques is necessary for detailed studies of complex microbial biogas consortia. Applying 16 different isolation strategies, bacterial and archaeal isolates were obtained from different mesophilic and thermophilic production- and laboratory-scale BGPs (Table 1). Furthermore, two archaeal members, namely *M. bourgensis* MS2^T^ [49] and *M. formicicum* MF^T^ [50], were obtained from the DSMZ and included in this study as the reference strains for methanogenic *Archaea* since they were also isolated from AD communities. German BGPs sampled for this study differed in utilized substrates ranging from maize silage, grass, and wheat straw to cattle and/or pig manure. Moreover, one digester analyzed was fed with organic residues and waste material as substrate. Additionally, a bio-waste compost treatment site close to the city of Munich (Germany) was sampled to isolate cellulolytic bacteria. Besides different renewable biomass sources utilized for the AD process, the biogas reactors differed regarding digester design, fermentation technology, and the applied temperature regime ranging from 37 to 54 °C.

This study comprises the analysis of 15 bacterial strains classified as belonging to the phyla *Firmicutes*, *Thermotogae*, and *Bacteroidetes* and seven archaeal isolates of the phylum *Euryarchaeota*. Details on all isolates of this study, their taxonomy, their origin, and the respective isolation strategy applied are provided in Table 1.

### Phylogenetic classification of the microbial isolates selected from different biogas communities

**Fig. 1:**
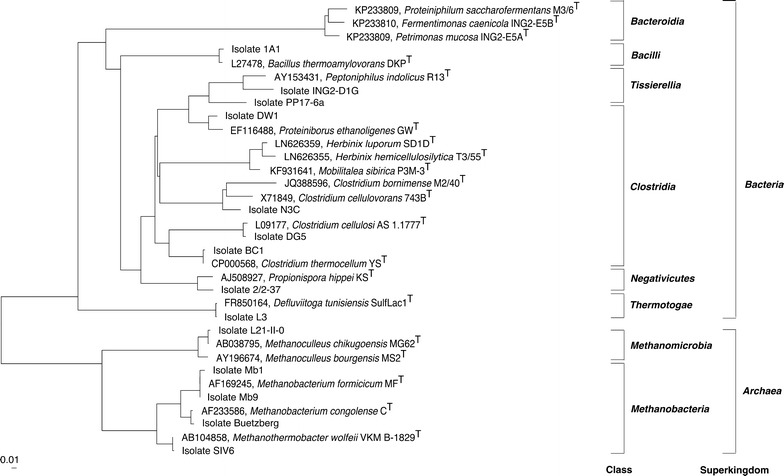
Phylogenetic diversity of archaeal and bacterial strains analyzed in this study in relation to the corresponding type species. The program ARB [35] was applied to construct the phylogenetic tree based on the full-length 16S rRNA gene sequences obtained from the strain’s genome sequences and in the case of closely related type species from the SILVA database [34]. The scale bar represents 1% sequence divergence

To determine the taxonomic position of the strains analyzed, their 16S rRNA gene sequences were compared to the corresponding sequences from closely related type strains deposited in the SILVA database (Fig. 1). The calculated phylogenetic tree comprises four main groups representing the phyla *Bacteroidetes*, *Firmicutes*, *Thermotogae*, and *Euryarchaeota*. Among the *Bacteroidetes* members, the strains *P. saccharofermentans* M3/6^T^, *P. mucosa* ING2-E5A^T^, and *Fermentimonas caenicola* ING2-E5B^T^ were recently described as novel species and were suggested to participate in hydrolysis and acidogenesis of the AD process [26].

Most of the bacterial strains analyzed were allocated to the phylum *Firmicutes*, and within this taxon to the classes *Clostridia*, *Bacilli*, *Tissierellia*, and *Negativicutes*. A diverse group of isolates belong to the class *Clostridia*. They are related to characterized species such as *Clostridium cellulosi* (also denominated as ‘*Ruminiclostridium*’ *cellulosi*), *Clostridium thermocellum* (also denominated as ‘*Ruminiclostridium*’ *thermocellum* [51], *Clostridium cellulovorans*, and *Clostridium bornimense*. The latter one was recently described as novel species [20]. All mentioned species represent lignocellulosic biomass degraders [20, 52, 53]. Two other *Clostridia* isolates, namely T3/55^T^ and SD1D^T^, were recently assigned to the species *Herbinix hemicellulosilytica* [54] and *Herbinix luporum* [55], respectively, of the new genus *Herbinix*. Both strains are distantly related to the type strain *Mobilitalea sibirica* P3M-3^T^ [56] and were described to be involved in thermophilic degradation of lignocellulosic biomass.

The isolates 1A1, ING2-D1G, and 2/2-37 are closely related to the species *Bacillus thermoamylovorans* (class *Bacilli*), *Peptoniphilus indolicus* (class *Tissierellia*), and *Propionispora hippie* (class *Negativicutes*), respectively. The corresponding reference strains were described to perform hydrolytic and acidogenic functions in the AD process [57–59].

Another isolate from a thermophilic BGP was classified as *D. tunisiensis* (phylum *Thermotogae*, class *Thermotogae*) representing an isolated branch of the bacterial part of the tree (Fig. 1). The strain *D. tunisiensis* L3 was described to be adapted to high temperatures and able to utilize different complex carbohydrates to produce ethanol, acetate, H_2_, and CO_2_ [27, 28]. The latter three metabolites represent substrates for methanogenic *Archaea*.

The strains *Sporanaerobacter* sp. PP17-6a and *Peptoniphilaceae* bacterium str. ING2-D1G are only distantly related to known bacterial species of the family *Clostridiales incertae sedis* and *Peptoniphilaceae* (90–91% identity), respectively, suggesting that they represent new species.

The fourth group of the phylogenetic tree represents methanogenic *Archaea* classified as members of the classes *Methanomicrobia* and *Methanobacteria* (both belonging to the phylum *Euryarchaeota*). Members of these classes were described to perform hydrogenotrophic methanogenesis utilizing CO_2_ and H_2_ as substrates for CH_4_ synthesis [18, 21].

### Genome sequence analyses of the whole set of microbial isolates selected

To gain insights into the functional potential of all strains listed in Table 1, their genomes were completely sequenced by application of HT sequencing technologies. Genome sequence information provides the basis for metabolic reconstruction and assignment of functional roles within the AD process, thus enabling biotechnological exploitation of genome features involved in fermentation processes utilizing renewable primary products.

Out of 22 genome sequences, nine, namely those of *Proteiniborus* sp. DW1, *Clostridium* sp. N3C, *Sporanaerobacter* sp. PP17-6a, *P. saccharofermentans* M3/6^T^, *P. mucosa* ING2-E5A^T^, *M. formicicum* Mb9, *M. congolense* Buetzberg, *M. wolfeii* SIV6, and *M. chikugoensis* L21-II-0, were newly established in this study. Genome sequences of the remaining 13 strains were published previously mainly in the form of Genome Announcements (for references, refer to Table 2). The genome sequences of the microorganisms analyzed were established on an Illumina MiSeq system. In silico and PCR-based gap closure strategies resulted in 13 finished and nine draft genome sequences. General genome features, e.g., genome structure, assembly status, size, GC content, and numbers of predicted genes, are summarized in Table 2. Established genomes range in size from 1.6 to 4.4 Mb and feature GC contents from 28.09 to 61.83%. Moreover, *C. bornimense* M2/40^T^, in addition to the chromosome, harbors a 699,161-bp chromid (secondary replicon) in its genome containing 680 coding sequences [37]. The methanogen *M. congolense* Buetzberg also harbors an accessory genetic element, namely a plasmid featuring a size of 18,118 bp. Genome annotation applying the GenDB 2.0 platform enabled functional interpretation of genes and reconstruction of metabolic pathways involved in the AD process. Genome analyses provided insights into the life-style and functional roles of bacterial and archaeal strains.

### Screening of the subset of bacterial genomes to identify genes encoding carbohydrate-active enzymes potentially involved in biomass degradation

**Fig. 2:**
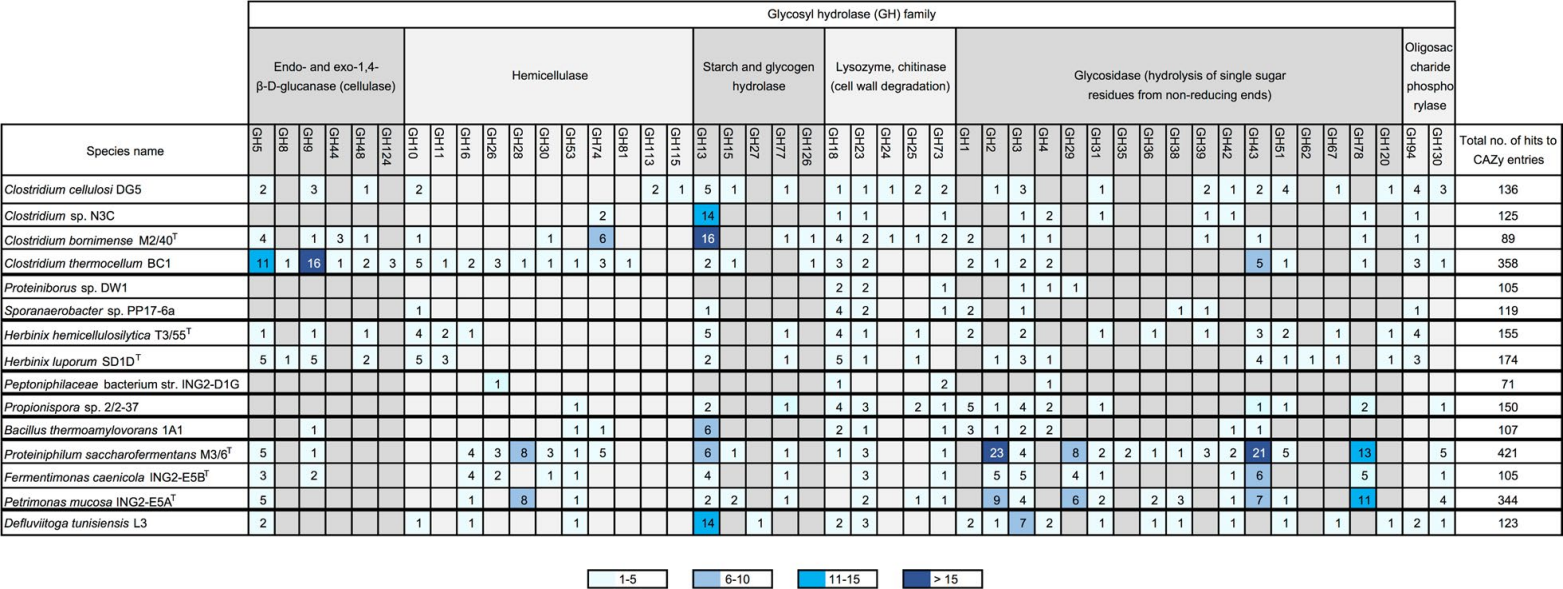
Diversity of genes encoding carbohydrate-active enzymes (CAZymes) predicted to be involved in hydrolysis and/or rearrangement of glycosidic bonds for each bacterial isolate studied. The screening for the presence of CAZymes was accomplished applying the HMM-based (Hidden-Markov-Model-based) carbohydrate-active enzyme annotation database dbCAN [40]. The numbers of bacterial genes belonging to a corresponding glycosyl hydrolase (GH) family are given in the fields

To elucidate genes encoding carbohydrate-active enzymes, functional genome annotation applying the HMM-based carbohydrate-active enzyme annotation database dbCAN [40] was performed (Fig. 2). Between 71 and 358 genes encoding enzymes or modules with predicted activity on carbohydrates were identified in each of the bacterial strains analyzed. Among them are dockerin-containing glycoside hydrolases (GH), representing putative cellulosomal enzymes, corresponding cohesin-containing scaffoldins, enzymes acting on large carbohydrate molecules, and carbohydrate-binding motifs involved in sugar binding. The obtained results separate the analyzed strains into two groups: group I strains were predicted to degrade cellulose and hemicellulose, whereas group II strains represent secondary fermentative bacteria relying on metabolites (mainly mono-, di-, and oligosaccharides) produced by group I members (as obvious presence of cellulolytic genes). The *Clostridiaceae* strains DG5, T3/55^T^, SD1D^T^, M2/40^T^, and BC1 harbor a more diverse repertoire of genes involved in the degradation of complex polysaccharides such as cellulose (GH5, GH8, GH9, GH48), xylan (GH10, GH11), and cellobiose- or cellodextrin-phosphorylase genes (GH94). Furthermore, genes for cohesin-containing putative scaffoldins and the corresponding dockerin-containing glycoside hydrolases with a potential for cellulosome formation were also identified in the genomes of these strains. Previous studies reported on the importance of the phylum *Firmicutes* for hydrolysis of cellulosic material in biogas digesters [12, 60]. In particular, *Clostridiaceae* and *Ruminococcaceae* members are involved in this first step of biomass digestion [11, 18]. *Clostridiaceae* strains *Proteiniborus* sp. DW1 and *Clostridium* sp. N3C were predicted to represent non-cellulolytic isolates (Fig. 2), whereas the cellulolytic strain *C. thermocellum* BC1 [61] is known to be a very efficient cellulose degrader since it encodes cellulosome components and is able to degrade hemicelluloses and pectins [60]. In contrast to the cellulolytic *Clostridiaceae*, the *Porphyromonadaceae* members, namely *P. saccharofermentans* M3/6^T^, *P. mucosa* ING2-E5A^T^, and *F. caenicola* ING2-E5B^T^, encode enzymes predicted to degrade pectins and a variety of hemicelluloses (GH16, GH26, GH28, GH30, GH53, GH74). These strains do not seem to be able to hydrolyze arabinoxylan (lack of GH10, GH11) and crystalline cellulose (lack of GH48). Likewise, *D. tunisiensis* L3 (*Petrotogaceae* family) also possesses a large set of genes predicted to facilitate cleavage of a variety of sugars including cellobiose, arabinosides (GH27), chitin (GH18), pullulan and starch (GH13), and lichenan (GH16) [28].

Another strain supposed to represent a secondary fermentative bacterium, namely *B. thermoamylovorans* 1A1 (*Bacillaceae* family), may contribute to oligosaccharide degradation with genes for GH1, GH2, GH3, or GH43 enzymes. In addition, genes required for growth on cellobiose are present in its genome. Considering the fact that strain 1A1 originally was isolated from a co-culture also containing *C. thermocellum* [61], it is assumed that *B. thermoamylovorans* 1A1 further metabolizes cellobiose produced by cellulolytic *Clostridia*.

Members of the genus *Propionispora* (*Veillonellaceae*) previously were identified in AD communities [62] and predicted to utilize mostly sugars and sugar alcohols, e.g., glucose, fructose, xylitol, or mannitol for growth [59]. The strain *Propionispora* sp. 2/2–37 analyzed in this study additionally harbors genes encoding enzymes participating in cellobiose, starch, and chitin degradation as determined by means of the CAZy analysis.

In contrast, the results obtained for *Peptoniphilaceae* bacterium str. ING2-D1G showed that this bacterium does not encode enzymes involved in the degradation of complex carbohydrates. However, the strain ING2-D1G encodes all enzymes needed to utilize amino acids and monomeric carbohydrates as a carbon source [22]. Its function in the anaerobic digestion process can be hypothesized to be associated with acidogenesis, which was supported by reconstruction of corresponding metabolic pathways.

### Prediction of fermentation pathways based on sequence information for the subset of bacterial genomes

**Fig. 3:**
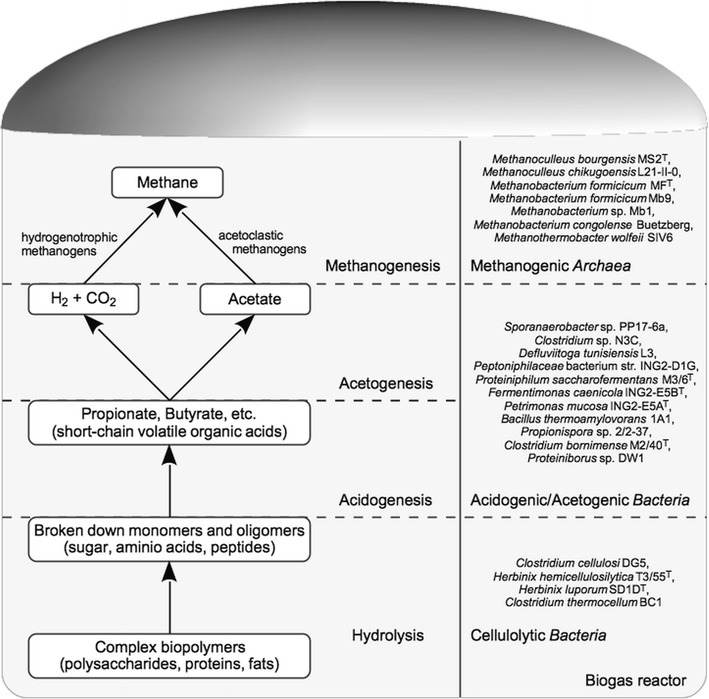
Overview of the four phases of the conversion of biomass into biogas and allocation of the analyzed microbial strains to the different conversion steps. Functional roles of the organisms were determined considering relevant KEGG pathways, namely the propionic acid, ethanol, formic acid, butyric acid, and lactic acid fermentation

Bacteria involved in AD perform a number of different fermentation pathways to recycle reduction equivalents that are produced in the course of metabolite utilization. To determine the fermentation type and the functional role of a given isolate within the biogas process, enzymes encoded in its genome were assigned to selected fermentation pathways represented in the KEGG database (Table 3, Additional file 2 and Fig. 3). Pathways leading to propionate, ethanol, formate, butyrate, acetate, and lactate synthesis were considered in this approach.

**Table 3:** Prediction of bacterial fermentation pathways as deduced from genome sequence information

Pathway analyzed	Predicted product after fermentation	*Clostridium cellulosi* DG5		*Clostridium* sp. N3C		*Clostridium bornimense* M2/40^T^		*Clostridium thermocellum* BC1		*Proteiniborus* sp. DW1		*Sporanaerobacter* sp. PP17-6a		*Herbinix hemicellulosilytica* T3/55^T^		*Herbinix luporum* SD1D^T^	
		GP	EP^a^	GP	EP	GP	EP^b^	GP	EP	GP	EP	GP	EP	GP	EP^c^	GP	EP^d^
Propionic acid fermentation^g^																	
Acrylyl-CoA pathway	Propionic acid	−	ND	−	NA	−	NC (D)	−	NA	+	NA	−	NA	−	NC (D)	−	N
Methylmalonyl-CoA pathway		−		−		−		−		−		+		−		−	
Ethanol fermentation	Ethanol	+	D	+		+	D	+		+		+		+	D	+	D
Formic acid fermentation																	
2,3-Butanediol fermentation	2,3-Butanediol	−	ND	−		−	ND	−		−		−		−	ND	−	ND
	Formic acid	−		+		+	D	+		−		+		+		+	
	CO_2_ and H_2_	−		−		+	D	+		−		−		−		−	
Mixed-acid fermentation	Ethanol	+	D	+		+	D	+		+		+		+	D	+	D
	Acetate	+		+		+	ND	+		+		+		+		+	
	Lactate	+	ND	+		+	D	+		+		−		+	ND	+	ND
	Succinate	−		+		+	ND	+		−		−		−		+	
Butyric acid fermentation	Butyrate	−		+		+	D	−		+		+		+	D	+	D
Homoacetogenesis	Acetate	+	D	+		+	ND	+		+		+		+		+	
Lactic acid fermentation																	
Homolactic acid fermentation	Lactate	+	ND	+		+	D	+		+		−		+	ND	+	ND
Heterolactic acid fermentation	Lactate	−		−		−	D	−		−		−		−		−	
	Acetate	+	D			+	ND	+		+		+		+	D	+	D
	Ethanol	+				+	D	+		+		+		+		+	

Pathway analyzed	Predicted product after fermentation	*Peptoniphilaceae bacterium* str. ING2-D1G		*Propionispora* sp. 2/2-37		*Bacillus thermoamylovorans* 1A1		*Proteiniphilum saccharofermentans* M3/6^**T**^		*Fermentimonas caenicola* ING2-E5B^**T**^		*Petrimonas mucosa* ING2-E5A^**T**^		*Defluviitoga tunisiensis* L3			
		GP	EP^a^	GP	EP^a^	GP	EP^a^	GP	EP^e^	GP	EP^e^	GP	EP^e^	GP	EP^f^		
Propionic acid fermentation^g^																	
Acrylyl-CoA pathway	Propionic acid	−	ND	−	D	−	ND	−	D	−	D	−	D	−	ND		
Methylmalonyl-CoA pathway		−		+		+		+		+		+		−			
Ethanol fermentation	Ethanol	−		+		+	D	+	ND	+	ND	+	ND	+			
Formic acid fermentation																	
2,3-Butanediol fermentation	2,3-Butanediol	−		+	ND	+	ND	−		−		−		−			
	Formic acid	−		+		+		+		+		+		−			
	CO_2_ and H_2_	−		−		−		−		−		−		+			
Mixed-acid fermentation	Ethanol	−		+	D	+	D	+		+		+		−			
	Acetate	+	D	+		+		+	D	+	D	+	D	+	D		
	Lactate	+	ND	+	ND	+	ND	+	ND	+	ND	+	ND	+	ND		
	Succinate	−		−		+		+		+		+		+			
Butyric acid fermentation	Butyrate	+	D	+	D	+		+		+		+	D	+			
Homoacetogenesis	Acetate	+	D	+		+	D	+	D	+	D	+		+	D		
Lactic acid fermentation																	
Homolactic acid fermentation	Lactate	+	ND	+	ND	+	ND	+	ND	+	ND	+	ND	+	ND		
Heterolactic acid fermentation	Lactate			−		−		−		−		−		−			
	Acetate	+	D	+	D	+	D	+	D	+	D	+	D	+			
	Ethanol	+	ND	+		+		+		+		+	ND	+			

Genomic loci encoding enzymatic functions participating to the corresponding fermentation type for each bacterial strain analyzed are listed in Additional file 2

+, synthesis of the corresponding fermentation end-product is predicted; −, pathway incomplete or misses key enzymes, the synthesis of the corresponding fermentation end-product is doubtful; EP, experimental proof; D, the corresponding fermentation product has been experimentally detected; GP, genes predicted applying metabolic reconstruction within the GenDB 2.0 system [39]; NA; not analyzed; NC, not confirmed; ND, fermentation product has been experimentally not detected

^a^Unpublished data

^b^Data published in [20]

^c^Data published in [54]

^d^Data published in [55]

^e^Data published in [26]

^f^Data published in [27]

^g^Pathways for propionic acid synthesis via succinate decarboxylation or amino acid degradation were not included

Certain bacteria are able to convert sugars, acids, alcohols, or amino acids to propionic acid under anaerobic conditions utilizing the methylmalonyl-CoA or the acrylyl-CoA pathways of the propanoate metabolism [27]. Among the analyzed bacteria, the strains *Propionispora* sp. 2/2-37, *P. saccharofermentans* M3/6^T^, *P. mucosa* ING2-E5A^T^, and *F. caenicola* ING2-E5B^T^ encode all enzymes of the methylmalonyl-CoA pathway for the production of propionic acid from pyruvate. Only the strain *Proteiniborus* sp. DW1 was predicted to utilize lactate for propionic acid production via the acrylyl-CoA pathway. Since the enrichment of propionic acid was described as an indicator for process imbalance [27, 63], data on the physiology of propionic acid-producing bacteria can be valuable for the optimization of the biogas plants.

Butyric acid-forming bacteria in biogas systems have been insufficiently characterized so far [27]. Genes encoding enzymes required for butyric acid formation via the butanoate pathway were found in the genomes of the strains *Propionispora* sp. PP16-6a, *Peptoniphilaceae* bacterium str. ING2-D1G, *C. bornimense* M2/40^T^, *P. saccharofermentans* M3/6^T^, *Clostridium* sp. N3C, *P. mucosa* ING2-E5A^T^, *F. caenicola* ING2-E5B^T^, and *B. thermoamylovorans* 1A1. Butanoate production was recently described for the strains *H. luporum* SD1D^T^ [55] and *H. hemicellulosilytica* T3/55^T^ [54]. However, the genomes of these bacteria only encode the last two enzymes of the butanoate pathway, namely the phosphate butyryl transferase Ptb and butyrate kinase Buk, predicted to be responsible for butanoate synthesis in these strains.

During acidogenesis, volatile organic compounds such as ethanol, acetate, and formate are produced in the course of the AD process. The latter two metabolites are substrates for methanogenic *Archaea.* Analysis of pathways involved in ethanol, acetate, and formate synthesis, i.e., the mixed-acid fermentation, revealed that all analyzed bacteria harbor genes encoding enzymes of this pathway (see Additional file 2). With the exception of the *Peptoniphilaceae* bacterium str. ING2-D1G, in all other isolates the necessary genes to produce ethanol from pyruvate were identified. Moreover, genes encoding enzymes participating in formate production were found in the *C. cellulosi* DG5, *C. bornimense* M2/40^T^, *D. tunisiensis* L3, *C. thermocellum* BC1, and *B. thermoamylovorans* 1A1 genomes. Furthermore, all analyzed bacteria were predicted to be able to produce acetate from acetyl-CoA. Genes encoding the enzymes phosphate acetyltransferase Pta (EC: 2.3.1.8) and acetate kinase Ack (EC: 2.7.2.1), converting acetyl-CoA to acetyl phosphate and subsequently to acetate, were found. In addition, genes encoding the enzymes pyruvate decarboxylase Pdc (EC: 4.1.1.1) and alcohol dehydrogenase Adh (EC: 1.1.1.1), converting pyruvate to acetaldehyde and finally to ethanol, were found in all genomes with the exception of the strain *Peptoniphilaceae* bacterium str. ING2-D1G, which does not possess an *adh* gene. Surprisingly, in the case of the strains *P. mucosa* ING2-E5A^T^, *F. caenicola* ING2-E5B^T^, and *P. saccharofermentans* M3/6^T^, no ethanol production was observed in growth experiments [26]. Possibly, the growth conditions tested might not be favorable to support ethanol synthesis.

Many bacterial species produce 2,3-butanediol under anaerobic conditions from glucose, with *Klebsiella oxytoca* and *Bacillus licheniformis* described as efficient 2,3-butanediol producers [64]. Among the bacteria analyzed, only *Propionispora* sp. 2/2–37 harbors a full set of genes encoding all necessary enzymes (refer to Additional file 2).

Lactic acid was found to be the main fermentation product from household waste digestion [65]. Members of the genera *Bacillus*, *Lactobacillus*, *Leuconostoc*, *Pediococcus*, and *Streptococcus* were previously described to produce lactic acid from several types of sugars [12, 47, 66]. To determine whether the analyzed bacteria have the potential to produce lactic acid, the genomes were screened for encoded enzymes involved in homolactic and heterolactic acid fermentation. With the expection of the strain *Sporanaerobacter* sp. PP17-6a, all other bacterial genomes were predicted to perform homolactic acid fermentation. They harbor all genes encoding necessary enzymes including the gene for lactate dehydrogenase Ldh (EC: 1.1.1.27) converting pyruvate to lactic acid. Furthermore, some genetic determinants of the heterolactic acid fermentation pathway were identified. However, none of the strains encodes a full set of the genes needed. Hence, the question which strains are responsible for lactic acid production remains unsolved.

### Prediction of methanogenesis pathways based on sequence information for the subset of archaeal genomes

The formation of CH_4_, the last step in the AD of biomass, is performed by methanogenic *Archaea* (Fig. 3). Based on their genetic repertoire, methanogens are able to perform either the hydrogenotrophic, acetoclastic, or methylotrophic pathway utilizing CO_2_ and H_2_, acetate, or methylamine and methanol, respectively, for CH_4_ production [67]. To predict the pathway by which the analyzed *Archaea* produce CH_4_, genes involved in the different methanogenesis pathways mentioned above were examined interpreting functional KEGG assignments calculated within GenDB (Table 4).

**Table 4:** Predicted genome features and traits of archaeal strains included in this study

Strain name	Features predicted						
	*Methanobacterium formicicum* MF^T^	*Methanobacterium formicicum* Mb9	*Methanobacterium* sp. Mb1	*Methanobacterium congolense* Buetzberg	*Methanothermobacter wolfeii* SIV6	*Methanoculleus bourgensis* MS2^T^	*Methanoculleus chikugoensis* L21-II-0
Methanogenesis-related hydrogenase genes encoded in the genome	*eha*, *ehb*, *frh*, *mvh*, *hdr*	*eha*, *ehb*, *frh*, *mvh*, *hdr*	*eha*, *ehb*, *frh*, *mvh*, *hdr*	*eha*, *ehb*, *frh*, *mvh*, *hdr*	*eha*, *ehb*, *frh*, *mvh*, *hdr*	*ech*, *frh*, *mvh*, *hdr*	*ech*, *frh*, *mvh*, *hdr*
Substrates used for methanogenesis	H_2_/CO_2_, F	H_2_/CO_2_, F	H_2_/CO_2_, F	H_2_/CO_2_, F	H_2_/CO_2_, F	H_2_/CO_2_, F	H_2_/CO_2_, F
Predicted metabolites required for growth	Acetate, cysteine^a^, vitamin B^a^	Acetate	Acetate	Acetate, lactate	Acetate	Acetate, lactate^b^	Acetate, lactate^b^

F, formate; H_2_, hydrogen; CO_2_, carbon dioxide

^a^Utilization of cysteine and vitamin B by the strain MF^T^ was described previously [50]

^b^No growth or methane production was detected on lactate for *Methanoculleus* species described previously [49, 82]

All *Archaea* analyzed encode a full set of genes involved in CH_4_ production from CO_2_ and H_2_. This result was as expected, as members of the families *Methanobacteriaceae* and *Methanomicrobiaceae* are known to solely perform hydrogenotrophic methanogenesis [68]. Additionally, genes for the formate dehydrogenase complex FdhA-B and a formate transporter FdhC for growth on formate as an alternative methanogenic substrate were identified in all seven analyzed genomes. For acetyl-CoA production from acetate, all seven genomes encode the acetyl-CoA synthetase Acs. Interestingly, methanogens from the genus *Methanoculleus*, namely the strains MS2^T^ and L21-II-0, also harbor a lactate dehydrogenase gene involved in conversion of lactate to pyruvate or vice versa. However, no growth or CH_4_ production from lactate has been described for the *Methanoculleus* species so far.

For activation of H_2_ during methanogenesis, all seven *Archaea* analyzed encode the cytoplasmic coenzyme F_420_-reducing [NiFe]-hydrogenases FrhA-D, the cytoplasmic [NiFe]-hydrogenase MvhADG, and the heterodisulfide reductase HdrABC in their genomes. The latter two enzyme complexes interact with the cytoplasmic [NiFe]-hydrogenase MvhADG, which was also identified in all investigated methanogens, for the coupled H_2_-driven reduction of ferredoxin and heterodisulfide CoM-S-S-CoB [69]. Furthermore, methanogens of the family *Methanobacteriaceae* encode the membrane-bound energy-converting [NiFe]-hydrogenases EhaA-T and EhbA-Q [70], whereas the *Methanomicrobiaceae* strains encode the energy-converting [NiFe]-hydrogenase EchA-F in their genomes. Members of the order *Methanomicrobiales* were described to exhibit a high affinity for H_2_ (ca. 0.1 µM resp. 15 Pa H_2_ pressure [71]), possibly providing an advantage over certain *Methanobacteriales* under conditions of low H_2_ partial pressure.

### Prevalence of bacterial and archaeal isolates in different microbial biogas communities analyzed by metagenome fragment mappings

To determine the prevalence or rather the abundance of the bacterial and archaeal isolates analyzed in this study in communities of production-scale BGPs, metagenome fragment mappings were done using deeply sequenced metagenomes from three mesophilic (BGP1-3) and one thermophilic (BGP4) agricultural BGPs which were published recently [41]. Configurations and process parameters corresponding to these BGPs are documented in the publication cited above. To identify metagenome sequence reads of the BGPs that match the genome sequences of the biogas isolates, these were mapped to the genomes applying Kallisto. Reads assigned to certain genomes were summed up and normalized according to dataset and genome sizes analogous to TPM (transcripts per million, [72]) values in RNASeq studies, to allow for quantitative comparisons.

Metagenome fragment mapping results were distinguished into the following groups: (I) abundant fully covered genomes, (II) less abundant but fully covered genomes, (III) rare but fully covered genomes, and (IV) rare, partially covered genomes (examples for each group are shown in Additional file 1).

**Fig. 4:**
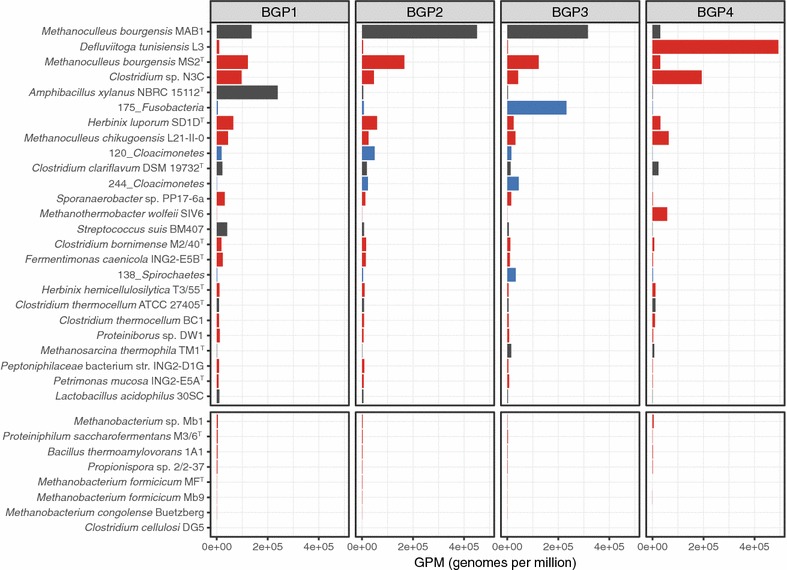
Prevalence of bacterial and archaeal strains within different biogas-producing microbial communities as determined by the fragment recruitment approach. Metagenome sequences derived from the microbial communities of three mesophilic (BGP1-3) and one thermophilic biogas plants (BGP4) described previously [41] were mapped on the genome sequences of the 22 strains analyzed in this study, the four MAGs described previously [41], and 46 publicly available genomes obtained from the RefSeq database [44]. Results for the 25 most abundant organisms are shown in the upper part of the figure. The prevalence of the remaining eight isolates of this study, representing non-abundant organisms, is shown in the lower part of the figure. The *x*-axis represents the number of GPMs (genomes per million; analogous to TPM = transcripts Per Million), and the *y*-axis shows the analyzed organisms. Isolates investigated within this study are shown in red, genome bins obtained from a previous study [41] in blue, and genomes obtained from the RefSeq database are visualized in black

Only three genomes, namely those of *Methanoculleus bourgensis* MS2^T^, *D. tunisiensis* L3, and *Clostridium* sp. N3C, fall into group I. *M. bourgensis* is abundant in all mesophilic BGPs studied and slightly less abundant in the thermophilic BGP, whereas *D. tunisiensis* and *Clostridium* sp. N3C are prominent in the thermophilic BGP (Fig. 4, Additional file 3).

Several of the analyzed strains were clearly detectable in the mesophilic BGPs but appeared to be only moderately abundant (group II). The strains *H. luporum* SD1D^T^, *M. chikugoensis* L21-II-0, *Sporanaerobacter* sp. PP17-6a, and *M. wolfeii* SIV6 fall into this category. They are supposed to perform functions that are also taken by other community members. In other words, the corresponding microbial guilds are composed of several species featuring similar functionalities. Specific adaptation of species within a guild may refer to slight fluctuations in environmental conditions with one or the other species being more competitive under a particular condition.

The strains *C. bornimense* M2/40^T^, *F. caenicola* ING-E5B^T^, *H. hemicellulosilytica* T3/55^T^, and *C. thermocellum* BC1 seem to be rare in most of the analyzed BGPs (group III), whereas the isolates *Proteiniborus* sp. DW1, *Peptoniphilaceae* bacterium str. ING-D1G, *P. mucosa* ING-E5A^T^, *Methanobacterium* sp. Mb1, *P. saccharofermentans* M3/6^T^, *B. thermoamylovorans* 1A1, *Propionispora* sp. 2/2-37, *M. formicicum* MF^T^, *M. formicicum* Mb9, *M. congolense* Buetzberg, and *C. cellulosi* DG5 seem to be, if at all, of minor importance in most BGPs (group IV).

Furthermore, the non-cultivable fractions of the biogas microbiomes residing in BGPs 1 to 4 were studied by Stolze et al. [41], applying metagenome assembly combined with a binning method. This approach enabled the identification of novel and uncharacterized species represented by MAGs, namely 206_*Thermotogae*, 175_*Fusobacteria*, 138_*Spirochaetes*, 244_*Cloacimonetes*, and 120_*Cloacimonetes*. To determine the prevalence of these  MAGs in the biogas microbiomes analyzed, fragment recruitments were performed. The obtained results showed that the species represented by the bin 175_*Fusobacteria* is abundant in the mesophilic BGP3, whereas both *Cloacimonetes* MAGs were abundant in BGP2 and BGP3. Furthermore, all three MAGs represent fully covered genomes and therefore fall into the groups I and II in the case of 175_*Fusobacteria* and both *Cloacimonetes* MAG, respectively. The bin 138_*Spirochaetes* is detectable in the mesophilic BGP3 but appeared to be only moderately abundant (group III). The MAG 206_*Thermotogae* is very similar to *D. tunisiensis* L3 showing an ANI (average nucleotide identity) value of 99.25%, indicating that these two members belong to the same species [73]. Fragment recruitments for such closely related microorganisms lead to random distribution of the corresponding metagenome sequences to both genome sequences resulting in underestimation of the abundances of both strains. Hence, the 206_*Thermotogae* MAG was not further considered for fragment recruitments.

Among the publicly available reference species, only the genomes of *M. bourgensis* MAB1 [74] originating from a laboratory-scale biogas reactor and *Amphibacillus xylanus* NBRC 15112 [75], isolated from compost of manure with grass and rice straw, were almost completely covered with metagenome sequences featuring high matching accuracy. The bacterial species *A. xylanus* NBRC 15112 was found to be highly abundant within the BGP1 microbiome, whereas the hydrogenotrophic methanogen *M. bourgensis* MAB1 was dominant in the mesophilic digesters 2 and 3 (Fig. 4). The genomes of both strains fall into group I regarding their fragment recruitment profiles. Among the microorganisms of group II, the species *C. clariflavum* involved in hydrolysis of cellulose and hemicellulose [76] and *Streptococcus suis* BM407, a human pathogen [77], were found to be nearly fully covered but less abundant.

Based on these findings, metagenome fragment mappings clearly showed that the culturomics approach led to isolation and characterization of dominant and therefore important members of the biogas microbiome. However, since it is assumed that many biogas community members cannot be cultured by currently available cultivation techniques, further prevalent key microorganisms remain to be discovered.

## Conclusions

Application of high-throughput and -*omics* technologies such as metagenomics, metatranscriptomics, metaproteomics, and genomics for the analysis of biogas microbial communities is becoming increasingly important. However, currently, the interpretation of generated data is limited due to the restricted availability of the corresponding and appropriate reference genome sequences connected with functional and metabolic information in public databases.

In this study, whole genome sequence information for 22 bacterial and archaeal strains was analyzed with respect to their metabolic functions in AD communities. For 15 bacterial strains, their participation in hydrolysis and/or acidogenesis/acetogenesis of plant biomass decomposition was predicted and partially verified by in vivo characterization of pure cultures. *Clostridium cellulosi* DG5, *H. hemicellulosilytica* T3/55^T^, *H. luporum* SD1D^T^, and *C. thermocellum* BC1 represent cellulose degraders, while the nine remaining bacteria presumably play a role in acidogenesis and/or acetogenesis. The seven analyzed methanogenic *Archaea* were predicted to produce CH_4_ via the hydrogenotrophic pathway, representing the final phase of the AD chain.

Among the microorganisms analyzed in this study, only two species, namely *M. bourgensis* and *D. tunisiensis*, were identified to play a dominant role within biogas microbial communities. *Defluviitoga tunisiensis* was proposed as a marker organism for the thermophilic biogas processes. This species is very versatile in the utilization of different sugars that can be converted to metabolites serving as substrates for methanogenesis. *Methanoculleus bourgensis* has frequently been found to dominate methanogenic sub-communities residing in production-scale BGPs and is assumed to be well adapted to high-osmolarity conditions and ammonia/ammonium concentrations prevailing when manure is used as a substrate for biogas production. Furthermore, the fragment recruitment analysis of MAGs published by Stolze et al. [41] could also show that in addition to the classical cultivation and isolation strategy, the metagenome assembly and binning approach may also enable the identification and characterization of previously unknown but abundant species featuring important functional potential in the context of the anaerobic digestion process.

It appeared that among the publicly available genomes only those of the species *A. xylanus*, *C. clariflavum*, and *C. thermocellum* were found to be well represented within biogas microbiomes, but do not reach the level of abundance as observed for *M. bourgensis* and *D. tunisiensis.* Surprisingly, among 5061 complete genome sequences archived in the public database NCBI, only those mentioned above seem to be of pronounced importance for agricultural biogas systems. Accordingly, the applied culturomics approach led to the isolation of further key AD species, thus providing genome sequence information for novel biogas community members. In the future, the non-cultivable fraction of AD communities should also be accessed by single-cell genomics to uncover genome sequence information of further, so far unknown biogas community members.

## Additional files

**Additional file 1.** Fragment recruitment of metagenome sequences derived from four biogas-producing microbiomes to the genome sequences of the exemplarily chosen strains *Amphibacillus xylanus* NBRC 15112^T^, *Clostridium* sp. N3C, *Fermentimonas caenicola* ING2-E5B^T^, *Methanobacterium formicicum* MF^T^ and *Methanoculleus bourgensis* MAB1. The *x*-axis: microbial genome analyzed, *y*-axis: percent identities of mapped metagenome reads.

**Additional file 2.** Genomic loci encoding enzymatic functions participating in the propionic acid, ethanol, formic acid, butyric acid and lactic acid fermentation for each strain analyzed.

**Additional file 3.** List of the 72 most abundant bacterial and archaeal strains within the biogas microbial communities analyzed, their GPM (genomes per million) values and further coverage statistics.

## Abbreviations

AD: anaerobic digestion
BGP: biogas plant
CCC: circulary closed chromosome
CAZymes: carbohydrate-active enzymes
CSTR: continuous stirred tank reactor
DSMZ: Leibniz Institute German Collection of Microorganisms and Cell Cultures
GH: glycosyl hydrolase
GPM: genomes per million
HT: high-throughput
KEGG: Kyoto Encyclopedia of Genes and Genomes
qPCR: quantitative ‘real-time’ polymerase chain reaction
TPM: transcripts per million
TRFLP: terminal restriction fragment length polymorphism
UASS: upflow anaerobic solid-state reactor
VFA: volatile fatty acids
