# Supplementary figures and images for: Genomics and prevalence of bacterial and archaeal isolates from biogas-producing microbiomes
Source: Biotechnol Biofuels. 2017 Nov 13;10:264. doi: 10.1186/s13068-017-0947-1 (PMC5684752; doi:10.1186/s13068-017-0947-1)

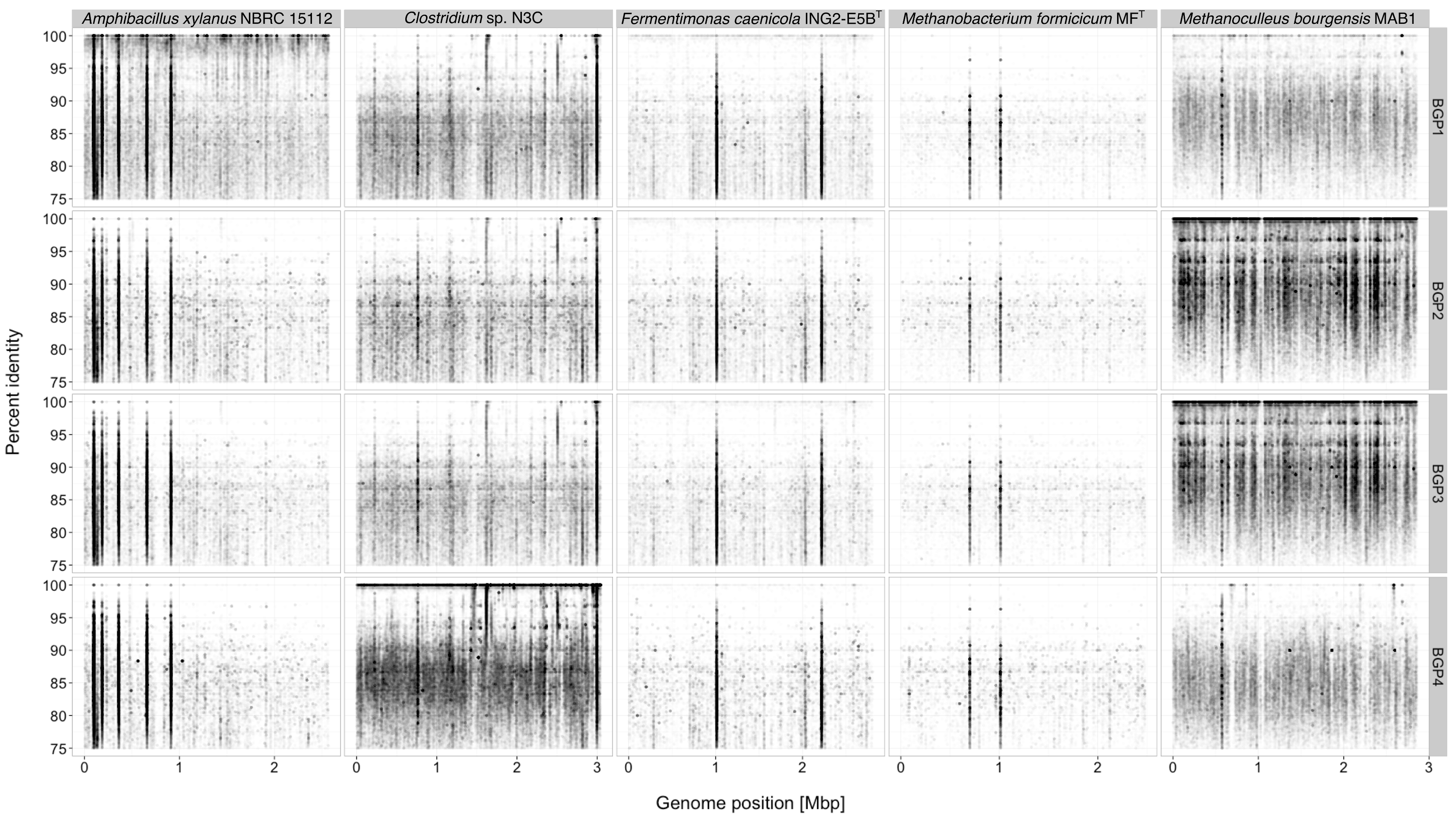

Supplement: Supplementary file 1 — Additional file 1. Fragment recruitment of metagenome sequences derived from four biogas-producing microbiomes to the genome sequences of the exemplarily chosen strains Amphibacillus xylanus NBRC 15112T, Clostridium sp. N3C, Fermentimonas caenicola ING2-E5BT, Methanobacterium formicicum MFT and Methanoculleus bourgensis MAB1. The x-axis: microbial genome analyzed, y-axis: percent identities of mapped metagenome reads. [file 13068_2017_947_MOESM1_ESM.tiff]
